# Safety of research bronchoscopy with BAL in stable adult patients with cystic fibrosis

**DOI:** 10.1371/journal.pone.0245696

**Published:** 2021-01-22

**Authors:** Daniel Aridgides, John Dessaint, Graham Atkins, James Carroll, Alix Ashare

**Affiliations:** 1 Section of Pulmonary and Critical Care Medicine, Dartmouth-Hitchcock Medical Center, Lebanon, New Hampshire, United States of America; 2 Department of Microbiology and Immunology, Geisel School of Medicine at Dartmouth, Hanover, New Hampshire, United States of America; Sapienza University of Rome, ITALY

## Abstract

Data on adverse events from research bronchoscopy with bronchoalveolar lavage (BAL) in patients with cystic fibrosis (CF) is lacking. As research bronchoscopy with BAL is useful for isolation of immune cells and investigation of CF lung microbiome, we sought to investigate the safety of bronchoscopy in adult patients with CF. Between November 2016 and September 2019, we performed research bronchoscopies on CF subjects (32) and control subjects (82). Control subjects were nonsmokers without respiratory disease. CF subjects had mild or moderate obstructive lung disease (FEV_1_ > 50% predicted) and no evidence of recent CF pulmonary exacerbation. There was no significant difference in the age or sex of each cohort. Neither group experienced life threatening adverse events. The number of adverse events was similar between CF and control subjects. The most common adverse events were sore throat and cough, which occurred at similar frequencies in control and CF subjects. Fever and headache occurred more frequently in CF subjects. However, the majority of fevers were seen in CF subjects with FEV_1_ values below 65% predicted. We found that CF subjects had similar adverse event profiles following research bronchoscopy compared to healthy subjects. While CF subjects had a higher rate of fevers, this adverse event occurred with greater frequency in CF subjects with lower FEV_1_. Our data demonstrate that research bronchoscopy with BAL is safe in CF subjects and that safety profile is improved if bronchoscopies are limited to subjects with an FEV_1_ > 65% predicted.

## Introduction

Flexible bronchoscopy is a versatile procedure used in Pulmonary Medicine to investigate and sample the airspaces as well as surrounding tissues. Bronchoscopy was first attempted by Killian in Germany during the late 1800s, followed by Chevalier Jackson in the United States [1]. This technique was improved upon by the invention of the flexible fiberoptic bronchoscope by Ikeda in Japan, allowing for investigation of more distal airways [1], followed by digital cameras placed on the end of flexible bronchoscopes. Flexible bronchoscopes contain a working channel, through which needles or forceps can be inserted to obtain biopsies of lung parenchyma, masses, or lymph nodes adjacent to the airways. Irrigation fluids and suction can also be applied through the working channel, and when the bronchoscope is wedged in a subsegmental airway they can be combined for a special technique termed bronchoalveolar lavage (BAL). BAL returns represent alveolar contents. In the healthy lung these consist primarily of alveolar macrophages [2]. In various disease states BAL can contain bacteria, fungi, as well as other immune cells [3–5].

Bronchoscopy with BAL is a very safe, minimally invasive procedure. Subjects are managed with conscious sedation consisting of low-dose opiates and benzodiazepines as well as topical anesthesia with lidocaine to the oropharynx and tracheobronchial tree. In many cases, topical anesthesia is sufficient and sedation is not required. Patients are provided supplemental oxygen and vital signs are monitored. During bronchoscopy, coughing is occasionally problematic but can generally be managed with opiates and lidocaine. Adverse events after bronchoscopy include hemoptysis, which can be present for up to 24 hours post-procedure. This is felt to be secondary to minor trauma from the bronchoscope, and occur in less than 1% of adult cases where biopsies are not performed [6]. Fever occurs in 2%-3% of bronchoscopies and is generally self-limited [7, 8]. Reactions against medications including allergies and exaggerated pharmacologic responses to sedatives are also possible. Methemoglobinemia secondary to lidocaine is a very rare example of a pharmacologic side effect [9]. Bronchospasm, refractory hypoxia, and pneumothorax are rare adverse events, with incidence rates well below 1% [6]. Overall mortality after flexible bronchoscopy is vanishingly rare and typically related to underlying chronic health conditions [6].

BAL in Cystic Fibrosis (CF) patients is sometimes used for microbiologic sampling when sputum cultures are not available. Historically, lavage was also performed for therapeutic purposes prior to development of modern airway clearance techniques [10, 11]. Sputum cultures are felt to contain the predominant pathogens present in the CF lungs [12–14], however the composition of less prevalent species within the lung microbiota is missed, as well as strain specific variation [12, 15, 16]. While throat swabs are often used for non-sputum producers to evaluate for common CF bacterial pathogens, we have previously shown that this test misses a significant number of *Pseudomonas* positive patients [17] and it cannot detect less prevalent species. In some centers, CF BAL is used in pediatric patients who cannot produce sputum [18], particularly for identification of early *Pseudomonas aeruginosa* infection [19]. Since the advent of triple modulator therapy [20–22], many patients are no longer producing sputum, however they are likely still colonized with bacteria as was seen in previous cohorts with mutations for which highly effective modulator therapy is available [23]. Bronchoscopy may be able to aid in the characterization of colonizing bacteria in these patients [24]. Specific risks to CF patients are mostly studied in children, and include a higher incidence of transient fever [25], however these are difficult to differentiate from early CF exacerbations that may have happened irrespective of bronchoscopy.

Bronchoscopy is used in research to purify and characterize immune cells from the alveolar space as well as to obtain microbiologic data [11, 12, 25–29]. To justify an elective invasive procedure for research with no direct clinical benefit the risks need to be minimal. Recent studies have evaluated the risk of research bronchoscopy in patients with COPD and asthma [30, 31], however little is known about the risks of research bronchoscopy in adults with CF. We report here the outcomes of our single-institution research bronchoscopy program on CF and non-CF volunteers with an excellent safety profile.

## Materials and methods

### Human subjects

All subjects were enrolled as part of an ongoing research study involving large volume BAL for isolation of primary lung macrophages to investigate differences between CF and non-CF primary lung macrophages. Written, informed consent was obtained for all subjects by a member of the study team. Healthy subjects, age 18–60, were enrolled if they were non-smokers and had no underlying cardiopulmonary or immunologic medical conditions. Subjects were excluded if they were on any immunosuppressive medication or if they had upper respiratory infection symptoms over the past 14 days. CF subjects, age 18–60, were enrolled if they had an FEV1 > 50% predicted, were not currently having an exacerbation, and were non-smokers. Subjects were excluded if they had been on oral or intravenous antibiotics over the past 28 days. All female subjects underwent a pregnancy test and were excluded if positive. This study was approved by the Institutional Review Board at Dartmouth Hitchcock Medical Center (#22781).

### Bronchoscopy

Following informed consent, all subjects underwent flexible fiberoptic bronchoscopy as previously described [12, 28]. Briefly, after local anesthesia with viscous lidocaine to the posterior pharynx and initiation of intravenous sedation, a bronchoscope was passed transorally through the vocal cords. BAL fluid was obtained from three subsegmental bronchi (five aliquots of 20 mL from each segment) for isolation of lung macrophages. CF subjects also had proximal airway mucus samples obtained via protected brush from two airway segments. Following the procedure, subjects were monitored per institutional protocol until they were stable for discharge.

### Statistical analyses

Statistical analyses were performed using GraphPad Prism (Prism, San Diego, CA).

## Results

Between November 2016 and September 2019, 32 bronchoscopies with BAL were performed on stable adult subjects with CF for isolation of primary lung macrophages as well as microbiome analyses. During the same time period, we performed 82 bronchoscopies with BAL on healthy adult volunteer subjects for isolation of primary lung macrophages. There was no significant difference between the CF and healthy cohorts with respect to age or sex although there was a trend toward more female subjects in the healthy control cohort (Table 1). CF subjects had an average FEV1 of 79.4%. Among the CF subjects, 87.5% had at least one copy of the F508del mutation, the most common disease causing mutation in the US, and 53% had two copies of the F508del mutation.

**Table 1:** Subject characteristics.

Characteristic	CF (n = 32)	HV (n = 82)	*P* Value
**Sex, female % (n)**	41% (13)	48% (39)	0.32
**Average age, years (SD)**	28 ± 4.9	29 ± 6.1	0.99
**FEV1, percent predicted (SD)**	79.4 ± 14		

Values are means ± standard deviation (SD); n = number of bronchoscopies; FEV1 = forced expiratory volume in 1 second; CF = cystic fibrosis; HV = healthy volunteer. P values determined by Fisher’s exact test.

Subjects’ heart rate, oxygen saturation, blood pressure, and end-tidal carbon dioxide were monitored continuously during bronchoscopy with a bedside monitor. To minimize potential adverse events, we excluded control subjects with any history of respiratory disease and CF subjects with severe airflow obstruction. Subjects were monitored in the endoscopy recovery area following the procedure until they were awake and alert and able to tolerate oral intake. A member of the research team contacted subjects 24 and 48 hours after the procedure to assess for adverse events and these phone calls were documented in the electronic medical record. Adverse events were graded as follows: 1) Grade 1 events include fatigue, nausea, sore throat, hoarseness, and cough; 2) Grade 2 events include fever, headaches, myalgias, and chest tightness; 3) Grade 3 events include hypotension requiring fluids or medication, prolonged hypoxia, or prolonged chest tightness; 4) Grade 4 events include hypotension unresponsive to medication or hypoxemic respiratory failure requiring mechanical ventilation. All adverse events were reviewed by an independent medical monitor. Throughout the duration of this study, no subjects have experienced Grade 3 or Grade 4 adverse events.

In 91% of healthy subject cases (n = 75), there were either no adverse events or mild Grade 1 adverse events. The most common adverse events among healthy subjects were sore throat and cough. Sore throat was reported by 21 (26%) and cough was reported by 19 (23%) of healthy bronchoscopy subjects (Table 2). Other common side effects include hoarseness and fatigue, which occurred in 6 (7%) and 10 (12%) of healthy subjects, respectively. Rarer side effects among healthy subjects include: fever (n = 4), headache (n = 3), nausea (n = 3), body aches (n = 2), dizziness (n = 1), and hemoptysis (n = 1). Among healthy subjects who reported fever within 48 hours of the procedure, one healthy subject also developed a productive cough with scant hemoptysis and was treated with a course of oral antibiotics. This Grade 2 adverse event resolved with antibiotic treatment and was reported to the IRB. All other reported side effects resolved within 48 hours of the procedure. No healthy subjects required hospitalization or emergency room treatment following research bronchoscopy with BAL. In follow up phone calls, the majority of healthy subjects reported no significant impact on their overall wellbeing and over 80% of subjects have opted to participate in the research bronchoscopy protocol multiple times.

**Table 2:** Adverse events.

	CF (n = 32)	HV (n = 82)	*P* Value
**Adverse Events % (n)**	59% (19)	50% (41)	0.26
Sore Throat	22% (7)	26% (21)	0.62
Cough	25% (8)	23% (19)	0.74
Hoarseness	9.3% (3)	7.3% (6)	0.79
Fatigue	13% (4)	12% (10)	0.99
**Fever**	**16% (5)**	**4.8% (4)**	**0.01**
**Headache**	**13% (4)**	**3.7% (3)**	**0.04**
Nausea	9.3% (3)	3.7% (3)	0.44

Values are percentage of total and (absolute number); n = number of subjects; CF = cystic fibrosis; HV = healthy volunteer. P values determined by Fisher’s exact test.

Of 32 CF bronchoscopies, 23 (72%) subjects reported either no adverse symptoms or mild Grade 1 adverse events within 48 hours of the procedure. Sore throat and increased cough occurred in 7 (22%) and 8 (25%) of CF subjects (Table 2) and were not significantly different from the numbers seen after healthy volunteer bronchoscopies. Although slightly less common, fatigue, nausea, and hoarseness occurred in 4, 3, and 3 CF subjects, respectively. Headaches occurred in 4 (13%) of CF subjects and this was significantly increased compared to the 3.7% seen in healthy volunteers. In all CF subjects, the headache resolved within 48 hours and did not require any intervention other than over the counter medication and oral hydration. Fever, with or without night sweats, was reported by 5 (16%) CF subjects within 48 hours of the bronchoscopy, which is significantly increased compared to the number of healthy volunteer subjects with fever. Among those 5 subjects who experienced fever, one was hospitalized for a pulmonary exacerbation within two weeks following the procedure. This subject had an FEV_1_ in the lower range (53%) of acceptable by our inclusion criteria prior to the procedure. An additional subject reported the onset of fevers approximately 7 days after the bronchoscopy and was hospitalized for a CF pulmonary exacerbation. However, a review by the independent medical monitor indicated that this hospitalization was not related to the procedure given the lack of symptoms in the first 48 hours following the procedure. No subjects required oral antibiotics or any other escalation of CF therapies. There were no significant differences noted in the rate of adverse events among CF subjects based on sex or age. However, 80% of CF subjects reporting fever and 75% of CF subjects reporting headache had an FEV_1_ < 65% predicted. In follow up phone calls, the majority of CF subjects reported that they did not experience an overall negative impact on their health. Over 50% of CF subjects have opted to participate in the research bronchoscopy protocol multiple times, suggesting no significant negative impact on their physical or mental wellbeing.

## Discussion

Patients with CF are chronically colonized with bacteria and it is standard of care to obtain sputum cultures for microbiologic assessment. However, only 30–40% of pediatric CF patients are able to routinely produce spontaneous sputum samples [32, 33]. Our prior work demonstrates that the use of throat swabs as a surrogate for sputum samples lacked sensitivity for the detection of *P*. *aeruginosa* [17]. Others have utilized BAL for periodic sampling as well as directed antibiotic therapy in pediatric CF patients [34]. While we do not routinely perform bronchoscopy for BAL in CF patients for clinical purposes, our research requires primary immune cells from the CF lung to investigate fundamental differences in airway inflammation in CF. In an investigation of research bronchoscopies performed in healthy volunteer subjects and CF subjects over a 34 months period, we found no significant difference in overall adverse events between CF and non-CF subjects. Specifically, there was no difference in reporting of sore throat, cough, hoarseness, fatigue, or nausea. CF subjects had an increased incidence of fever and headache and we reported one CF subject who initially reported a fever and subsequently required hospitalization that was likely related to the procedure.

Research bronchoscopy with BAL is used for isolation of primary immune cells, evaluation of cellular and microbial contents, and airway examination. While research protocols often involve performing bronchoscopy on healthy volunteers without underlying lung disease, to investigate the pathophysiology it is often necessary to obtain clinical samples via BAL from human subjects with underlying lung disease. A recent study investigated the safety of research bronchoscopy in patients with COPD and asthma [30]. The results of this study include safety data of bronchsocopy with BAL as well as protected brush sampling and endobronchial biopsies. In this study of 239 subjects, they reported increased dyspnea among COPD subjects compared to control subjects. In addition, a prior study found that investigative bronchoscopy in severe asthmatics was well tolerated [31].

Recent advances in CF, including the development of highly effective modulator therapy for the majority of patients, have resulted in increased longevity and quality of life for people living with CF [35]. Despite these advancements, chronic lung inflammation remains a problem in CF. In fact, a survey of patients and families living with CF identified the need for better anti-inflammatory and anti-infective therapies in CF [36]. To identify novel therapeutic targets to combat CF lung inflammation, the use of primary immune cells is critical. Therefore, research bronchoscopy with BAL is a highly useful tool to facilitate the development of improved therapies to target inflammation. Ours is the first study to specifically investigate the safety of research bronchoscopy in adults with CF. In addition, studies of CF lung microbiology may be more challenging by decreased sputum production after initiation of highly effective modulator therapy. Despite the lack of sputum, bacteria and fungi remain in the lung, and bronchoscopy may be the only way to interrogate this for both clinical and research purposes.

While CF patients tolerated research bronchoscopy similarly to the healthy control cohort, we did find an increase in fevers and had one hospitalization in the CF group. These findings occurred almost exclusively in CF patients with lower lung function. Reduction in FEV_1_ has been shown to correlate with degree of inflammation [37], suggesting that the subjects with fever and hospitalization may have increased lung inflammation. We hypothesize that the mobilization of bacteria and inflammatory cells may cause the release of cytokines and LPS, resulting in fevers. This is consistent with the fact that CF subjects with relatively normal lung function rarely experienced fever.

Our study demonstrates that bronchoscopy with BAL can be safely performed in CF subjects for research purposes. We found a similar rate of side effects between CF and non-CF subjects, with more side effects occurring in CF subjects with worse lung function. To optimize the safety profile of research bronchoscopies, while still allowing performance of the procedure to obtain primary immune cells of samples for microbiome analyses, we recommend limiting the procedure to CF patients with an FEV_1_ of greater than 65% predicted.
